# MoCap-Referenced Neck–Shoulder sEMG–IMU Decoding for Discrete Assistive Commands: A Pilot Study

**DOI:** 10.3390/s26134027

**Published:** 2026-06-25

**Authors:** Ameer H. Majeed, Farah Masood, Hussein A. Abdullah

**Affiliations:** 1 Biomedical Engineering Department, Al-Khwarizmi College of Engineering, University of Baghdad, Baghdad 17635, Iraq; 2 College of Engineering, University of Guelph, Guelph, ON N1G 2W1, Canada; habdulla@uoguelph.ca

**Keywords:** sEMG, IMU, MoCap, assistive devices, neck–shoulder muscles

## Abstract

Hands-free command interfaces are essential for users who cannot reliably operate joysticks or upper-limb myoelectric control. Neck–shoulder surface electromyography (sEMG) is a promising alternative; however, performance is often reported using window-level validation which can overestimate accuracy due to overlap and trial leakage, and false-trigger behavior is not always quantified when an idle REST state is included. This pilot study presents a motion-capture (MoCap)-referenced decoding framework that uses four bilateral upper trapezius (UT) and sternocleidomastoid (SCM) sEMG channels with integrated inertial measurement units (IMUs). Optical MoCap was used as an external kinematic reference to support baseline-posture assessment and movement-execution quality control. Seven commands were decoded (shrug L/R, double shrug, rotation L/R, rotation + shrug L/R). To enable an eight-class formulation, a REST class was defined using low-activity segments extracted from baseline recordings and included in the evaluation. Computationally efficient time-domain sEMG features, pattern/symmetry descriptors, and baseline-referenced IMU kinematics (including an SCM yaw-range indicator) were classified using linear discriminant analysis (LDA), k-nearest neighbors (kNN), and linear support vector machine (SVM), evaluated using within-subject testing, trial-wise grouped cross-validation, and leave-one-subject-out (LOSO) testing. Across six participants, within-subject mean best-per-subject accuracy was 96.02% (seven-class) and 96.35% (eight-class); and pooled trial-wise accuracy reached 92.1% and 90.5%, respectively. Under LOSO, best-configuration accuracy decreased to 60.4% and 63.8% for the seven-class and eight-class formulations, respectively. Across the top LOSO configurations, REST FAR ranged from approximately 9.8% to 25.6%. These findings demonstrate controlled offline pilot feasibility and quantify key generalization and REST false-activation trade-offs, providing a foundation for future validation in larger, more diverse, and clinically relevant populations.

## 1. Introduction

Some users cannot rely on joysticks or upper-limb myoelectric control because of amputation, high-level spinal cord injury, or severe upper-limb dysfunction. For these users, hands-free command interfaces may provide an alternative way to control assistive devices. Surface electromyography (sEMG) is one possible input signal because it records voluntary muscle activity non-invasively and with high temporal resolution. It has been used in prosthetic and robotic end effectors, powered wheelchairs, and wearable assistive devices [1,2,3]. In many systems, short analysis windows are used to extract low-latency features, which are then mapped to discrete commands using conventional feature-based machine learning methods. Reviews of sEMG feature extraction and sEMG–IMU fusion for pattern recognition indicate that lightweight feature-based methods can achieve good classification performance with low processing requirements, supporting their use in future wearable real-time systems [1,2].

Although sEMG-based assistive control has advanced in recent years, important practical and methodological limitations remain. EMG recordings can be affected by skin–electrode contact, motion artifacts, fatigue, anatomical differences between users, and day-to-day variation, which may reduce stability outside controlled laboratory settings [1,2,4,5,6]. Electrode reattachment and small placement shifts are especially important concerns as they can change the sampled motor-unit activity and affect the distribution of commonly used features [7]. Another concern is how classification accuracy is evaluated. In sliding-window analysis, neighboring windows from the same repeated trial are often highly similar. If windows from the same trial appear in both the training and test data sets, the model may be tested on data that are too close to the training data, producing an overly optimistic estimate of performance. A further limitation is that idle-state behavior is often not reported. For discrete command interfaces, false commands during rest can be problematic in mobility applications, so including a REST class and reporting false activations are important for evaluating safety-related performance.

When upper-limb muscles are unavailable or difficult to activate reliably, the neck and shoulder region can provide an alternative source of control. The sternocleidomastoid (SCM) and upper trapezius (UT) are superficial muscles, which support their use for non-invasive surface recording, and they are involved in simple voluntary movements such as head rotation and shoulder elevation. Previous work has shown that neck EMG and head-orientation signals can be used as command sources for assistive interfaces [8]. More recent neck–shoulder EMG studies have also shown that several discrete commands can be classified using a limited number of SCM/UT channels with time-domain features and classical classifiers [9,10,11]. For example, Flower and Poonguzhali reported a dual-movement neck–shoulder EMG approach that classified nine control commands using three channels, supporting the feasibility of compact neck–shoulder EMG interfaces for assistive mobility control [9]. However, the literature also points to remaining challenges, particularly the gap between high subject-dependent performance and reliable generalization to unseen users, as well as the effect of recording variability [1,7,9].

Wearable head-motion sensing has also been investigated outside the specific context of the present study. Severin proposed a head-posture monitoring system based on three inertial sensors to classify posture-risk levels and provide real-time feedback for healthcare/posture-correction applications [12]. More recently, Zawalski et al. investigated artificial-neural-network detection of minimal motion patterns for assistive-device control in patients with a high level of disability [13]. In addition, Sugiarto et al. compared sEMG and high-density EMG, combined with IMU input, for head-orientation prediction in a virtual-reality application [14]. Together, these studies establish wearable head-motion and neck-muscle sensing as active research areas, while leaving a specific gap for compact bilateral UT/SCM sEMG–IMU decoding of discrete assistive commands with REST false-activation analysis, leakage-aware validation, LOSO testing, and MoCap-referenced movement-execution quality control.

Multimodal sensing can be useful because EMG and IMU signals describe different aspects of the same movement. EMG mainly reflects muscle activation, while IMU data describes the resulting motion. This is helpful when two commands have similar EMG amplitudes but different head or shoulder movement patterns. Previous reviews have shown that combining sEMG and IMU features can improve recognition performance in some tasks, although the improvement depends on the selected sensors, the movement type, and the validation method [1]. In the present study, optical MoCap was not used as a classifier input or as ground truth for user intention. Instead, it was used as an external kinematic reference to support baseline-posture assessment, movement-execution checking, and trial-quality control. This distinction is important because high classification accuracy alone does not confirm that all recorded trials were performed consistently or with the intended movement direction. Optical MoCap is widely used in biomechanics for marker-based kinematic assessment [15], but in the present study it was used only as an external quality-control reference rather than as classifier input. This study decoded discrete neck–shoulder commands from four bilateral sEMG channels over the UT and SCM muscles. The same probes also recorded IMU data. The command set included seven voluntary movements: left/right shrug, double shrug, left/right rotation, and left/right rotation-plus-shrug. An additional REST class was built from low-activity baseline segments to evaluate an eight-class setting. The EMG feature set included time-domain features, cross-channel pattern descriptors, and bilateral symmetry measures to describe signal amplitude, side-to-side differences, and muscle recruitment patterns. IMU features were referenced to the baseline posture. One of these features was SCM yaw range, which was used to help separate pure shrug commands from commands that also involved rotation. MoCap was used to check baseline stability and movement execution only; it was not included in the classifier input. We expected subject-specific decoding to outperform cross-subject decoding when models were tested on unseen participants. Therefore, performance was evaluated using within-subject testing, trial-wise grouped cross-validation on pooled data, and leave-one-subject-out (LOSO) testing, with conventional window-level pooled cross-validation included as a reference comparison. In the eight-class analysis, REST false activation rate (FAR) was used to quantify idle windows that were misclassified as movement commands, in addition to reporting overall accuracy. This evaluation was used to separate subject-dependent performance from unseen-user performance and to show the effect of adding REST on both accuracy and false activations.

The contribution of this pilot study is the integrated evaluation of a compact bilateral UT/SCM sEMG–IMU command-decoding framework using MoCap-referenced quality control, leakage-aware trial-wise validation, LOSO testing, and REST false-activation analysis.

This study makes the following contributions:

- MoCap is incorporated as an external reference to review trial quality without being used as a classifier input.
- The evaluation includes trial-wise grouped cross-validation to provide a less optimistic estimate than conventional window-level splitting.
- Cross-subject generalization is quantified using LOSO testing, providing a more realistic estimate of performance for unseen users.
- The eight-class setting includes REST FAR to quantify how often idle windows are incorrectly classified as movement commands, supporting deployment-oriented assessment.
- EMG-only, IMU-only, EMG+IMU, and EMG+IMUfilter modes are compared using computationally efficient features and classical classifiers relevant to future real-time implementation.

The remainder of this paper is organized as follows. Section 2 describes the experimental protocol, instrumentation, preprocessing, feature extraction, and evaluation designs. Section 3 reports the results of the within-subject, pooled trial-wise, and LOSO evaluations for the seven-class and eight-class settings. Section 4 discusses the findings in relation to previous studies and presents the main limitations and future directions. Section 5 concludes the paper.

## 2. Materials and Methods

### 2.1. Study Design and Participants

This pilot study presents an experimental protocol, data collection process, and offline analysis pipeline for decoding discrete neck–shoulder commands from surface electromyography (sEMG) recorded over the bilateral upper trapezius and sternocleidomastoid muscles. Six healthy male adult participants (S01–S06; age: 21.0 ± 2.4 years, range: 18–25 years) were included. None of the participants reported current neck or shoulder pain, neuromuscular disorders, or previous neck/shoulder injury or surgery that could affect task performance. Integrated inertial measurement units (IMUs) were recorded concurrently with sEMG, and optical motion capture (MoCap) was used as an external kinematic reference for movement-execution quality control. The analyses compared EMG-only, IMU-only, EMG+IMU, and EMG+IMUfilter feature sets for both a seven-class formulation and a REST-augmented eight-class formulation. Performance was evaluated using within-subject testing, leakage-controlled trial-wise grouped cross-validation on pooled data, and leave-one-subject-out (LOSO) testing. All preprocessing, feature extraction, and analyses were performed in MATLAB R2024b (MathWorks, Natick, MA, USA). The study was conducted in accordance with the Declaration of Helsinki and approved by the Scientific Committee of the Biomedical Engineering Department, Al-Khwarizmi College of Engineering, University of Baghdad. Written informed consent was obtained from all participants. All participant data were anonymized using subject codes before analysis. EMG, IMU, and MoCap files were stored using these coded identifiers, and no directly identifying information was used in the analysis files. MoCap data were used for marker-based kinematic analysis and quality control; no video-based identification was used in the classification pipeline.

### 2.2. Instrumentation

Surface EMG and inertial data were acquired using a DUE+ wireless bipolar system (OT Bioelettronica, Torino, Italy), consisting of a desktop sync-station/charger and wireless bipolar probes. Four probes were used to record bilateral signals from the upper trapezius (UT) and sternocleidomastoid (SCM) muscles (UT_L, UT_R, SCM_L, SCM_R). Each probe incorporated an integrated nine-axis IMU, consisting of a triaxial accelerometer, triaxial gyroscope, and triaxial magnetometer. The IMU was configured in NDOF fusion mode and provided absolute orientation as quaternions (W, X, Y, Z), where W is the scalar component and X, Y, and Z are the vector components. EMG signals were sampled at 2000 Hz per channel, while IMU quaternions were updated at 100 Hz. In the exported files, EMG and IMU data were stored on a shared 2000 Hz time base, resulting in repeated quaternion values between IMU updates [16].

**Figure 1:**
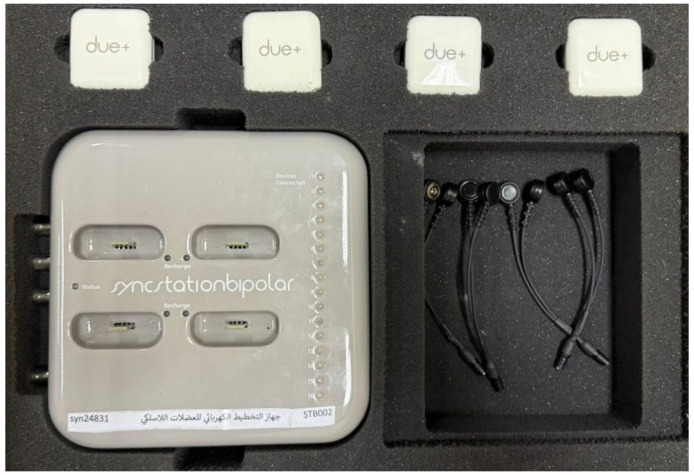
OT Bioelettronica DUE+ wireless sEMG acquisition system (OT Bioelettronica, Italy). The sync station/charger is shown on the left, and the wireless bipolar probes with electrode leads are shown on the right. The non-English label on the device identifies the wireless EMG acquisition system.

Head and upper-body movements were recorded concurrently using a multicamera infrared optical motion-capture system controlled by 3DMA software 2023.0 (OT) software, version 2023.0 (STT Systems, San Sebastián, Spain). Marker positions were sampled at 100 Hz and exported for offline analysis. MoCap was used only as an external kinematic reference for movement-execution quality control and was not used as classifier input. Figure 1 shows the DUE+ acquisition system and wireless probes.

### 2.3. Sensor Placement and Marker Configuration

Four bipolar sEMG probes were placed bilaterally over the upper trapezius (UT) and sternocleidomastoid (SCM) muscles, forming four recording channels: UT_L, UT_R, SCM_L, and SCM_R. Electrode placement followed SENIAM recommendations for superficial muscles. The bipolar axis was aligned with the main muscle fiber direction. Before electrode placement, the skin was cleaned with alcohol, and hair was removed when needed to reduce impedance and motion artifacts.

For the UT, probes were positioned over the muscle belly approximately halfway between the acromion and the C7 region to capture shoulder elevation activity while limiting cross-talk. For the SCM, probes were positioned over the muscle belly along the line between the mastoid region and the sternoclavicular area, avoiding tendon insertions. Reference electrodes were placed over stable bony landmarks, including the acromion and clavicle, to improve signal stability during head and shoulder movements. Because each sEMG probe contained an integrated IMU, inertial measurements were co-located with the EMG recording sites. IMU orientation was referenced to the baseline posture by computing baseline-referenced kinematic descriptors. This reduced sensitivity to sensor mounting orientation and supported consistent interpretation across trials.

**Figure 2:**
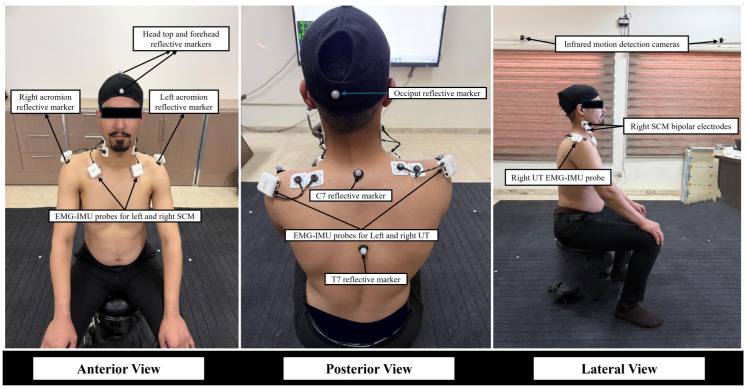
Experimental setup and sensor placement. Anterior, posterior, and lateral views show the EMG–IMU probes positioned bilaterally over the upper trapezius (UT) and sternocleidomastoid (SCM) muscles, together with the reflective markers used for optical motion capture (MoCap). MoCap served as an external kinematic reference for movement-execution quality control and was not used as classifier input.

Reflective markers were used to track head, neck, and upper-body movement for movement-execution quality control. Markers were placed according to the STT Systems cervical spine seven-point analysis protocol [17]. The seven markers were positioned on the head top, forehead/head front, occipital/head back, C7 vertebra, right acromion, left acromion, and T7 vertebra. Marker trajectories were inspected for missing data and flatline segments. MoCap was used as an external reference for quality control, including confirmation of baseline stability, checking gross movement direction such as left/right rotation, and identifying trials with poor kinematic data. Thus, MoCap remained a quality-control modality rather than part of the decoding feature set. The experimental setup, probe placement, and reflective marker configuration are shown in Figure 2.

### 2.4. Experimental Protocol and Command Set

Each participant completed a structured recording session that included two baseline recordings (BL1 and BL2), four maximum voluntary contraction (MVC) trials, and repeated trials of the target command set. All recordings were performed while the participant was seated in a neutral starting posture. Participants were instructed to keep the trunk as still as possible, return to the neutral head and neck position before each repetition, and avoid unnecessary torso movement during command execution. This controlled seated posture was used to reduce variability during the pilot feasibility protocol.

Baseline recordings were collected while participants maintained a relaxed neutral seated posture. These recordings were used to estimate baseline noise, select the cleaner baseline for calibration, and construct REST samples for the REST-augmented formulation. To reduce transient effects at the start of recording, baseline quality metrics were calculated over a consistent interval from 2 to 15 s.

MVC trials were recorded to provide channel-specific normalization references. MVC recordings were obtained using resisted contractions. For the upper trapezius channels, participants performed resisted shoulder elevation while holding a weight that could be sustained for the required MVC contraction period. For the sternocleidomastoid channels, participants performed resisted neck rotation while the experimenter applied manual resistance against the attempted rotation. These MVC recordings were processed to obtain robust reference values for each channel, which were then used to calculate percent MVC (%MVC) features during offline analysis. Functional MVC values derived from the command trials were not used because the command trials were the target data for classification; using their maximum values for normalization could make the scaling task-dependent and may introduce information from the evaluated movement data into feature scaling.

Before the experimental command recordings, participants were familiarized with the command set through verbal instructions and an instructional video containing visual and audio cues. Participants then completed a short practice period to ensure that each movement was understood and performed consistently.

**Figure 3:**
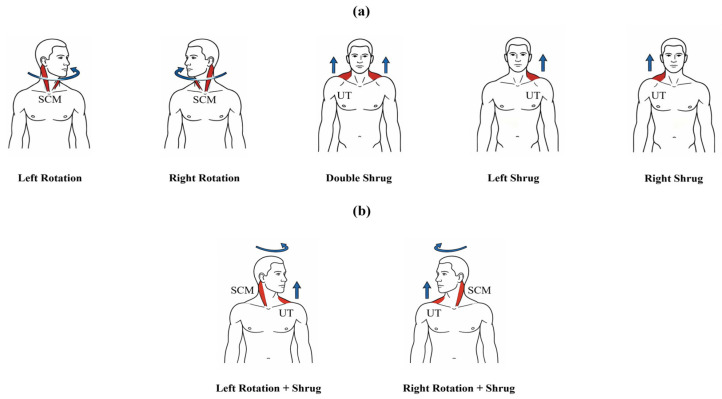
Schematic illustration of the adopted neck–shoulder command set used during data acquisition. (**a**) Single-axis commands: rotation left/right, shrug left/right, and double shrug. (**b**) Compound commands: left/right rotation-plus-shrug. Left/right are defined from the participant’s perspective. Red regions indicate primary muscles (UT, upper trapezius; SCM, sternocleidomastoid).

**Figure 4:**
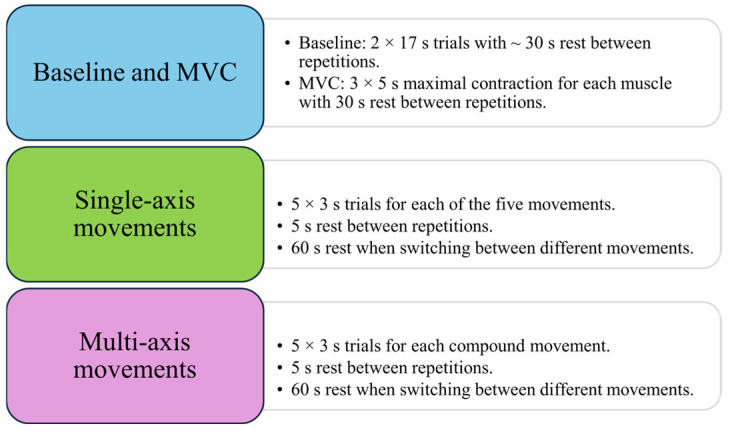
Experimental protocol and session timing. The diagram summarizes baseline and MVC recordings, single-axis movement trials, compound movement trials, repetition counts, and rest intervals.

After baseline and MVC recordings, participants performed seven commands: left shrug, right shrug, double shrug, left rotation, right rotation, left rotation-plus-shrug, and right rotation-plus-shrug. The command classes were recorded in a structured order rather than a randomized order to keep the pilot recording procedure simple and consistent across participants. Each command was repeated across several trials, with short rest periods between repetitions to reduce fatigue and support consistent performance. sEMG, IMU, and MoCap data were recorded simultaneously for all trials. Figure 3 illustrates the seven decoded commands, while Figure 4 summarizes the experimental timing and session structure.

**Figure 5:**
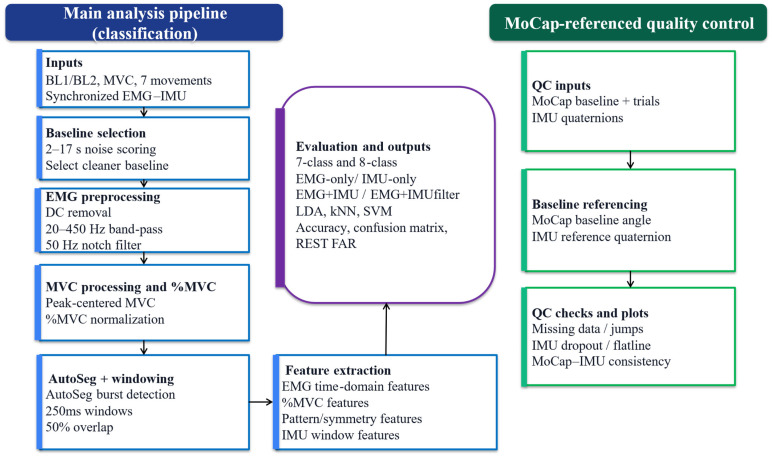
Overview of the analysis workflow. The main classification pipeline (left) summarizes baseline selection, EMG preprocessing, MVC-based normalization, windowing/AutoSeg, and feature extraction for EMG-only, IMU-only, EMG+IMU, and EMG+IMUfilter modes under seven-class and REST-augmented eight-class formulations. The MoCap-referenced quality-control branch (right) uses baseline-referenced MoCap and IMU signals to assess data integrity, including missing data, flatlines, and MoCap–IMU consistency, and to support trial-execution quality control.

In addition to the seven-class formulation, a REST-augmented eight-class formulation was evaluated. REST samples were constructed from low-activity baseline data. Baseline recordings were divided into 2 s blocks, and only low-activity blocks were retained to reduce dependence between neighboring REST windows. Analysis windows were then extracted from the retained REST segments using the same windowing scheme applied to the movement trials. The full processing and evaluation workflow, including baseline selection, feature extraction, REST construction, and validation design, is summarized in Figure 5.

### 2.5. Windowing and Artifact-Robust Segmentation

**Figure 6:**
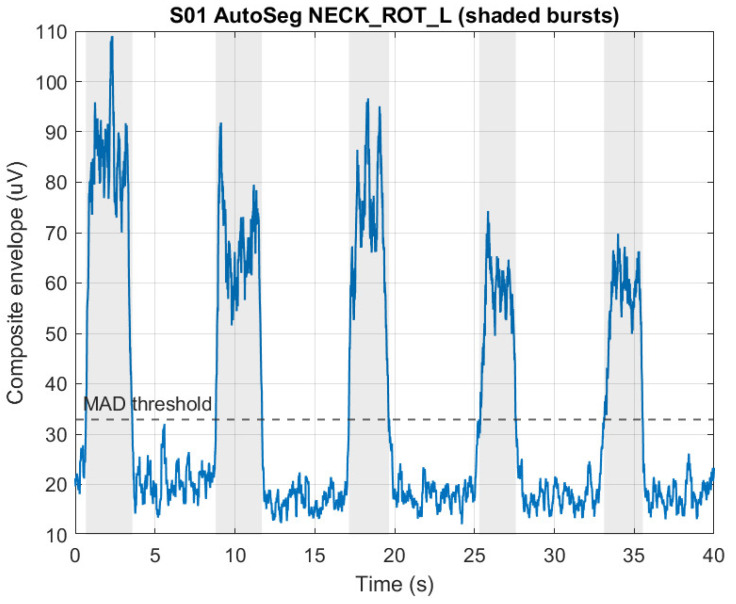
Automatic repetition segmentation (AutoSeg) using the composite EMG RMS envelope, defined as the maximum RMS across the four channels. The dashed line denotes the robust MAD-derived detection threshold, and the shaded regions indicate the five detected repetitions used for window-level feature extraction after discarding the first 2 s of the recording.

Signals were segmented into fixed-length 250 ms windows with 50% overlap, corresponding to a hop size of 125 ms, for feature extraction and classification. This window length was selected to balance responsiveness for future real-time use with stable estimation of time-domain features. To reduce the effect of sudden spikes and motion-related outliers, robust spike limiting was applied within each window using the median and median absolute deviation (MAD). MAD was used because it provides a robust estimate of signal variability and is less affected by transient spikes than standard deviation-based thresholds. This procedure constrained extreme values while preserving the EMG waveform needed for feature calculation. The present pipeline was evaluated offline. Although the selected window length, update interval, time-domain features, and classical classifiers are compatible with future real-time implementation, embedded computational benchmarking and online closed-loop testing were not performed in this pilot study. All subsequent EMG and IMU features were calculated using the same window boundaries to maintain temporal alignment across modalities. Figure 6 shows the RMS-envelope-based trial segmentation and repetition detection used for quality checking.

### 2.6. Signal Preprocessing and Alignment

#### 2.6.1. EMG Unit Handling and DC Offset Removal

Before filtering, each EMG channel was converted to microvolts (µV). As exported files can contain values in V or mV and may not always be labeled consistently, signal magnitude and header patterns were checked, and consistent scaling was applied when required to obtain values in µV. A DC offset was then removed by subtracting the sample mean from each channel.

#### 2.6.2. EMG Filtering

EMG signals were filtered using zero-phase forward–backward filtering to avoid phase distortion; therefore, the filtering step did not introduce a causal time delay in the EMG signal. A 50 Hz infinite impulse response (IIR) notch filter was applied to reduce mains power-line interference, followed by a fourth-order Butterworth band-pass filter (20–450 Hz) to suppress low-frequency motion artifacts, baseline drift, and high-frequency noise. The filtered signals were then used for windowing and feature extraction.

#### 2.6.3. IMU Preprocessing and Baseline-Referenced Orientation

The 3D orientation of each IMU probe was recorded as a unit quaternion. Each quaternion sample was normalized to unit length. For each sensor, a baseline reference quaternion was computed by averaging the quaternions over the baseline interval (2–15 s) and normalizing the result. Relative orientation was then computed with respect to baseline using quaternion multiplication. The scalar component of the relative quaternion was used to calculate the angle magnitude based on the shortest-rotation convention. Angular speed was estimated as the absolute value of the time derivative of this angle.

A baseline-adaptive motion threshold was set for each sensor, as described in Equation (1). Percentile-based thresholds are commonly used as data-driven gates in movement-sensing pipelines [18]. The multiplier k is a conservative heuristic that can be adjusted depending on the required detection sensitivity. In this study, k=3 was chosen to reduce false motion flags during baseline while preserving sensitivity to intended movement.

(1) $$T_{\omega ,s}=kP_{95}\left(\omega_{\mathrm{base},s}\right),\;k=3$$

where s indexes the IMU sensor/probe, $P_{95}(\cdot )$ is the 95th percentile of the baseline angular-speed distribution, and $T_{\omega ,s}$ is the sensor-specific motion threshold. This threshold was used to create a binary indicator for each window, allowing meaningful motion to be distinguished from baseline drift.

#### 2.6.4. Window-Level Alignment Between EMG and IMU

EMG features were extracted using the fixed sliding windows described in Section 2.5. Although the exported files used the 2000 Hz EMG time base, IMU quaternions were updated at 100 Hz and therefore appeared as repeated values between updates. During preprocessing, repeated quaternion entries were removed by retaining only rows where the quaternion changed, allowing IMU features to be computed at the effective 100 Hz update rate. For each EMG analysis window, the retained IMU samples falling within the same time interval were selected, and IMU features were computed over that interval. When no IMU samples were available within a given window, the corresponding IMU feature values were set to zero to preserve a fixed feature-vector length across all windows and subjects. Because IMU features were baseline-referenced, these zero entries were treated as missing/neutral values rather than additional motion information.

#### 2.6.5. Yaw-Based Rotation Indicator

To represent rotation content within the analysis windows, a yaw-based rotation indicator was derived from the SCM IMU sensors. First, each SCM quaternion was expressed relative to its baseline reference orientation. The baseline-referenced relative quaternions were then converted to Euler angles using a ZYX rotation sequence, where yaw was taken as the rotation about the local z-axis of the baseline-referenced sensor frame. This yaw signal was converted to degrees and unwrapped over time to avoid discontinuities.

For each analysis window, the yaw range was computed separately for SCM_L and SCM_R. The final rotation indicator was defined as the maximum SCM yaw range within the window. This feature was used as a relative axial-rotation descriptor rather than an absolute anatomical head-yaw measurement. It was intended to help distinguish rotation-containing commands from shrug-only commands, especially when UT activation dominated the EMG pattern.

#### 2.6.6. MoCap Baseline Referencing for Quality Control

**Figure 7:**
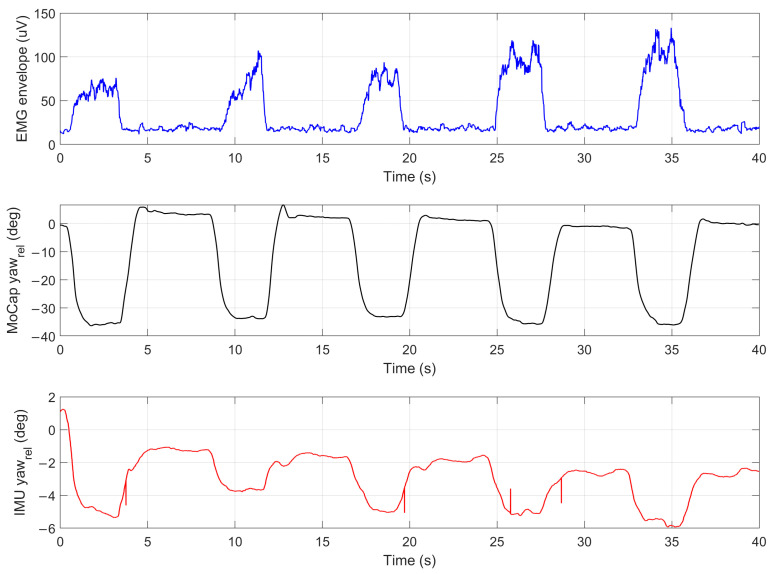
Tri-modal quality-control example for a representative trial (S01, right cervical rotation). The EMG, MoCap, and IMU traces are shown on the same post-trim trial time base after removal of the initial MoCap calibration segment and the predefined initial EMG/IMU discarded segment used in movement analysis. **Top**: smoothed EMG composite RMS envelope. **Middle**: baseline-referenced MoCap cervical axial rotation. **Bottom**: baseline-referenced IMU yaw from the selected SCM probe. The aligned trajectories illustrate cross-modal consistency for trial-quality control and are not intended as a physiological onset-latency analysis.

For MoCap-referenced quality control (QC), kinematic signals were baseline-referenced using the same calibration baseline interval (2–15 s). This allowed movement directionality and magnitude to be compared consistently across trials and participants. An example of cross-modal agreement between the EMG envelope, baseline-referenced MoCap cervical axial rotation, and baseline-referenced IMU yaw from the selected SCM sensor is shown in Figure 7. Because the IMU probes were mounted on local muscle sites, IMU yaw was interpreted as a relative rotation indicator rather than an absolute anatomical MoCap-equivalent angle. For the quality-control visualization, temporal alignment followed the predefined acquisition and preprocessing protocol. The MoCap recording included an initial calibration segment before the movement portion; therefore, the first 10 s of the MoCap file were removed before plotting. EMG/IMU movement files were processed using the predefined 2 s initial discard used throughout the movement analysis. After these trimming steps, the EMG/IMU and MoCap time vectors were re-zeroed to the same post-trim trial time base and truncated to the same analysis duration. No additional post hoc time shifting was applied for the visualization. EMG preprocessing used zero-phase forward–backward filtering; therefore, the filtering step did not introduce a causal phase delay. The EMG trace in Figure 7 represents a smoothed composite RMS envelope and is shown for qualitative quality control rather than physiological onset-latency analysis.

### 2.7. Feature Extraction

For each analysis window (Section 2.5), features were computed from the preprocessed signals described in Section 2.6. The feature vector included time-domain sEMG features for each channel, cross-channel pattern and bilateral symmetry descriptors derived from the four-channel activation profile, and IMU kinematic features computed using the same window boundaries. The feature set was kept computationally efficient to support future real-time implementation.

#### 2.7.1. Time-Domain sEMG Features (Per Channel)

For each channel and analysis window, standard time-domain features were extracted from the filtered and spike-limited EMG signal. These features included root mean square (RMS), mean absolute value (MAV), waveform length (WL), zero crossings (ZC), and slope sign changes (SSC). To account for amplitude differences across participants and channels, RMS was also expressed as a percentage of the channel-specific maximum voluntary contraction reference (%MVC), as described in Section 2.4. The %MVC feature was calculated using Equation (2):

(2) $$\%MVC=100\cdot \frac{RMS}{MVC+\varepsilon }$$

where RMS is the root mean square value of the EMG window, MVC is the recommended channel-specific maximum voluntary contraction reference, and ε is a small constant used to avoid division by zero.

#### 2.7.2. Cross-Channel Pattern Descriptors

To describe how activation was distributed across UT, SCM, and the left/right sides, cross-channel pattern descriptors were computed from the four-channel %MVC activation vector. These descriptors included the total activation sum (pSum), normalized channel proportions (pNorm), and left–right ratio terms for UT and SCM. The pNorm features represented each channel’s share of the total activation, whereas the left–right ratios were used to capture laterality during unilateral and rotation-related commands.

#### 2.7.3. Bilateral Symmetry and Coupling Features

Bilateral structure was represented using symmetry and coupling descriptors computed separately for UT and SCM. These features quantified the balance between the left and right sides and indicated whether activation within each muscle pair was mainly unilateral or bilateral. They complemented the cross-channel pattern descriptors by supporting discrimination of laterality-dependent commands.

#### 2.7.4. IMU Window Features and Motion Screening

IMU features were computed per sensor within each EMG analysis window using baseline-referenced signals (Section 2.6). Kinematic descriptors included window-level angle range and variability, along with angular-speed summary measures such as mean and 95th-percentile angular speed. A binary motion-screening indicator was derived for each sensor by comparing the window angular speed with the baseline-derived threshold. This indicator was used as a motion-presence cue during feature extraction and for the EMG+IMUfilter analysis, where it flagged windows containing movement-related IMU activity relative to baseline.

#### 2.7.5. Rotation Indicator Feature

A yaw-based rotation indicator was added to represent axial rotation content within each window. This feature was calculated as the maximum SCM yaw range within the window, as described in Section 2.6.5. It was included to support discrimination between shrug-only commands and commands that involved rotation, especially in compound movements where UT activation may dominate the EMG amplitude pattern.

### 2.8. Classification, Feature-Set Modes, Validation Designs, and Performance Metrics

#### 2.8.1. Feature-Set Modes

Four analysis modes were evaluated to examine the contribution of sEMG and inertial information and the effect of IMU-based window screening. The EMG-only mode used only sEMG-derived features. The IMU-only mode used only baseline-referenced IMU window features, including the SCM yaw-range rotation indicator described in Section 2.6.5. The EMG+IMU mode combined sEMG features with IMU window features derived from baseline-referenced inertial signals, including the SCM yaw-range rotation indicator. The EMG+IMUfilter mode used the EMG feature set after retaining movement windows that satisfied the IMU motion-screening criterion. This criterion was based on the baseline-derived angular-speed thresholds described in Section 2.6.3. For the eight-class formulation, REST windows were retained to allow calculation of REST false activation rate.

#### 2.8.2. Classifiers

Three conventional multiclass classifiers were evaluated: linear discriminant analysis (LDA), k-nearest neighbors (kNN), and linear support vector machine (SVM). LDA was implemented using MATLAB’s discriminant classifier, *fitcdiscr*. The kNN classifier used K=5 neighbors and was implemented using *fitcknn* with *NumNeighbors* = 5. The linear SVM was implemented using an error-correcting output codes (ECOC) approach with linear learners, using *fitcecoc* with *templateLinear*. All models were trained and tested using the same data splits and metrics to allow direct comparison across methods and class settings.

#### 2.8.3. Feature Standardization

Z-score standardization was applied using statistics calculated from the training data only. For each split or fold, the training-set mean vector μ and standard deviation vector σ were computed. Training features were transformed as (X − μ)/σ, and the same μ and σ were then applied to the corresponding test set. Near-zero standard deviations were replaced with a small constant to avoid division instability.

#### 2.8.4. Validation Designs

Four validation designs were used to assess subject-specific performance, conventional pooled performance, leakage-controlled pooled performance, and cross-subject generalization:

- Within-subject evaluation: Models were trained and tested separately for each participant to estimate subject-specific decoding performance. Trial/repetition grouping was used so that windows from the same repetition were kept within the same fold.
- Window-level stratified fivefold cross-validation on pooled data: Windows from all participants were pooled and divided into five stratified folds based on class labels. This design was included as a conventional comparison, but it can provide optimistic estimates when overlapping windows from the same trial are split between training and test folds.
- Trial-wise grouped stratified fivefold cross-validation on pooled data: Windows were pooled across all participants, but windows sharing the same trial identifier were kept within the same fold. This reduced leakage caused by overlapping windows within the same trial while maintaining class balance across folds. The design remained subject-dependent because data from the same participants could still appear in both training and test sets.
- Leave-one-subject-out (LOSO) evaluation: Models were trained on N−1 participants and tested on the held-out participant. This process was repeated until each participant served once as the test subject.

For the REST-augmented eight-class setup, REST windows were included to quantify idle-state misclassification using REST FAR. No test-set balancing was applied, so the reported test metrics reflected the original class distribution.

#### 2.8.5. Performance Metrics

Performance was summarized using accuracy, reported as mean ± standard deviation across folds for cross-validation designs and across held-out participants for LOSO. Confusion matrices were used to examine class-level error patterns. For the REST-augmented eight-class setup, the REST false activation rate (FAR) was additionally calculated as the proportion of REST windows misclassified as any movement class, as shown in Equation (3):

(3) $$FAR_{REST}=\frac{N_{FA}}{N_{REST}}$$

where $N_{FA}$ is the number of REST windows predicted as movement commands, and $N_{REST}$ is the total number of REST windows. REST FAR was reported alongside accuracy to reflect safety-relevant idle-state behavior.

## 3. Results

### 3.1. Within-Subject Performance

Within-subject decoding showed high accuracy in both class settings (Table 1). Across the six participants, the mean best-per-subject accuracy was 96.02% for the seven-class formulation and 96.35% for the REST-augmented eight-class formulation. The highest within-subject accuracies were 98.77% for the seven-class formulation and 99.29% for the eight-class formulation. These results show that the selected time-domain sEMG features and cross-channel descriptors can separate the command classes effectively when subject-specific calibration data are available.

These high accuracies should be interpreted in the context of the controlled same-session protocol, the homogeneous healthy participant group, and the clearly distinguishable command set.

### 3.2. Subject-Dependent Pooled Evaluation with Trial-Wise Grouping

**Figure 8:**
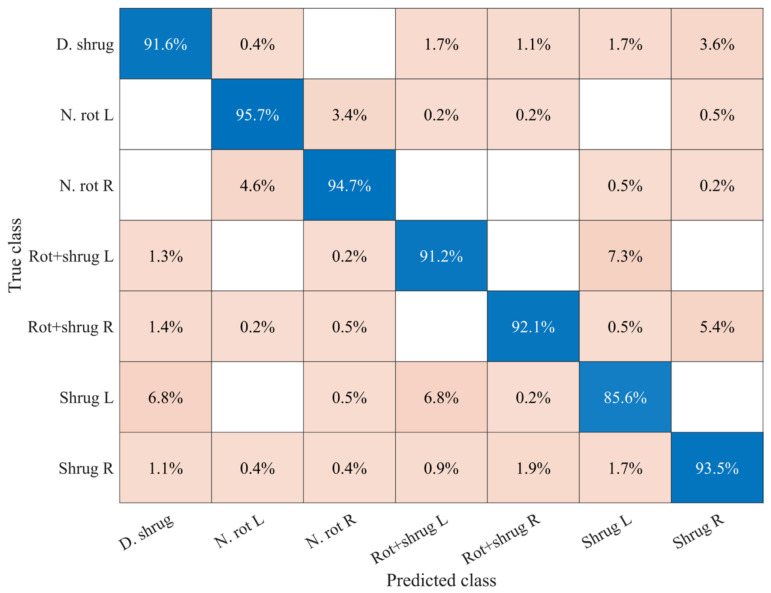
Row-normalized confusion matrix for the best-performing subject-dependent pooled trial-wise grouped cross-validation configuration in the seven-class formulation. The best configuration used EMG+IMU features with kNN. D. shrug denotes double shoulder shrug, and N. rot denotes neck rotation. Values are row-normalized percentages. Blue cells indicate correct classifications along the diagonal, whereas orange cells indicate off-diagonal misclassifications; color intensity reflects the row-normalized percentage.

Subject-dependent pooled evaluation was performed using two fivefold cross-validation designs: conventional window-level stratified cross-validation and trial-wise grouped cross-validation (Table 2). The window-level design gave higher accuracies, reaching 96.92 ± 0.55% for the seven-class formulation and 97.08 ± 0.33% for the REST-augmented eight-class formulation. However, this estimate can be optimistic because overlapping windows from the same trial may be split between training and test folds. With trial-wise grouping, all windows from the same trial were kept within the same fold. Under this design, the best pooled accuracies were 92.08 ± 1.05% for the seven-class formulation and 90.51 ± 1.93% for the eight-class formulation. For the pooled eight-class setting, REST FAR was 0.00 ± 0.00 for the best-performing configurations under both validation designs. The trial-wise grouped design remains subject-dependent, but it provides a more conservative pooled estimate than window-level splitting. Therefore, the trial-wise grouped results were emphasized over conventional window-level splitting when interpreting subject-dependent pooled performance. The row-normalized confusion matrix for the best trial-wise grouped seven-class configuration is shown in Figure 8.

To make the effect of feature-set mode and classifier choice more explicit, Table 3 summarizes the main configuration-level results across EMG-only, IMU-only, EMG+IMU, and EMG+IMUfilter modes using LDA, kNN, and SVM classifiers. This comparison was included to show whether performance was dependent on a specific sensing mode or classifier, rather than relying only on averaged best-case summaries.

Taken together, the comparison shows that kNN performed best in the subject-dependent trial-wise grouped evaluation, whereas SVM was more consistent under LOSO testing. The added IMU-only analysis provided a pure kinematic reference condition. IMU-only performance was lower than the corresponding EMG-only and combined EMG+IMU configurations in both trial-wise grouped CV and LOSO testing, indicating that inertial information alone did not explain the main classification performance. Therefore, IMU-derived features should be interpreted as complementary to the sEMG feature set rather than as a replacement for muscle-activation information. The lower LOSO accuracies across modes indicate that cross-subject generalization remained more challenging than same-session subject-dependent decoding.

### 3.3. Cross-Subject Generalization (LOSO)

**Figure 9:**
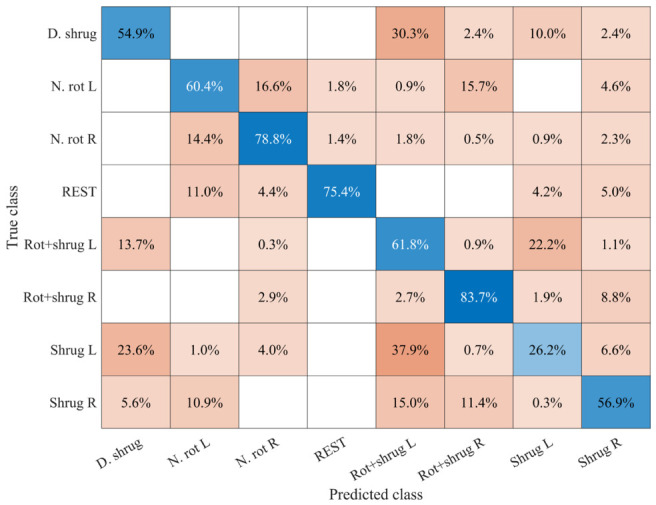
Row-normalized confusion matrix for the best-performing leave-one-subject-out (LOSO) configuration in the REST-augmented eight-class formulation. The best configuration used EMG+IMUfilter with linear SVM. D. shrug denotes double shoulder shrug, and N. rot denotes neck rotation. Values are row-normalized percentages. Blue cells indicate correct classifications along the diagonal, whereas orange cells indicate off-diagonal misclassifications; color intensity reflects the row-normalized percentage.

Cross-subject generalization was evaluated using leave-one-subject-out (LOSO) testing, as shown in Table 4. Under LOSO, the best configuration achieved 60.43 ± 5.41% accuracy for the seven-class formulation and 63.78 ± 9.06% accuracy for the REST-augmented eight-class formulation. In both settings, the best-performing configuration was EMG+IMUfilter with linear SVM. For the eight-class formulation, REST FAR was 24.58 ± 37.03%, indicating that false activations during idle periods varied substantially across held-out participants. These results show that cross-subject decoding was more challenging than within-subject and pooled subject-dependent evaluation. This reduction supports interpreting LOSO as a more conservative estimate of unseen-user performance and highlights the need for subject-specific calibration, adaptive normalization, or user-specific fine-tuning in future assistive-control studies. Figure 9 presents the row-normalized confusion matrix for the best LOSO configuration in the REST-augmented eight-class setting.

### 3.4. REST False Activation and the Accuracy–Safety Trade-Off

**Figure 10:**
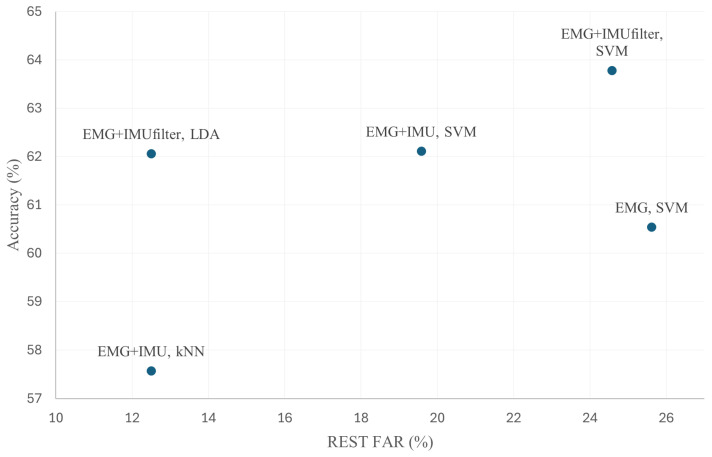
Accuracy versus REST false activation rate (FAR) for the top five LOSO configurations in the REST-augmented eight-class formulation. Each point represents a mode–classifier configuration. The figure illustrates that the configuration with the highest accuracy did not necessarily produce the lowest REST FAR.

In the REST-augmented eight-class formulation, REST false activation rate (FAR) varied across LOSO configurations. Across the top configurations, REST FAR ranged from 9.8% to 25.6%, showing that higher classification accuracy did not always correspond to lower false activation during rest. The configuration with the highest LOSO accuracy did not necessarily produce the lowest FAR, indicating that accuracy and REST FAR should be considered together when selecting a deployment-oriented model. Figure 10 presents the accuracy–FAR trade-off for the top LOSO configurations. Therefore, accuracy and REST FAR were interpreted together rather than using accuracy alone to select a deployment-oriented configuration.

### 3.5. Ablation of the SCM Yaw-Range Indicator

To examine whether the SCM yaw-range indicator was a dominant contributor to classification performance, an ablation analysis was performed by removing this feature from the EMG+IMU configurations. Table 5 summarizes the effect of this removal under trial-wise grouped cross-validation and LOSO testing. The effect was minor and configuration-dependent. In trial-wise grouped cross-validation, removing SCM yaw-range changed EMG+IMU kNN accuracy from 92.08% to 91.70% in the seven-class setting and from 90.51% to 90.21% in the REST-augmented eight-class setting. Under LOSO testing, the eight-class EMG+IMU SVM configuration changed from 62.11% to 61.48%, while the best EMG+IMUfilter SVM configuration remained unchanged at 63.78%. These findings indicate that SCM yaw-range provided supportive kinematic information in some configurations but was not the dominant driver of classification performance.

## 4. Discussion

### 4.1. Context and Main Interpretation

Neck and shoulder signals have been investigated as alternative command sources when hand or upper-limb function is limited, using neck EMG, head/neck orientation, or a combination of both for assistive control [8]. Recent neck–shoulder EMG work has also supported the feasibility of classifying movement commands for mobility-assistive control scenarios [9]. The present pilot study extends this context by showing that high subject-specific decoding performance can be achieved using compact time-domain and pattern-based features, while cross-subject generalization remains more difficult. Within-subject accuracy exceeded 96% in both the seven-class and REST-augmented eight-class formulations, whereas LOSO accuracy decreased to approximately 60–64%. This difference indicates that subject-specific calibration captures participant-dependent activation patterns more effectively than models trained on other participants. The results also show that REST false activation behavior should be considered alongside accuracy, because configurations with higher accuracy did not always produce lower REST FAR. However, these findings should be interpreted as controlled offline pilot feasibility in a small homogeneous healthy cohort, rather than as evidence of immediate clinical or real-time assistive-device readiness.

### 4.2. Feature Choice and Within-Subject Decoding

Within-subject performance was consistently high for both the seven-class and REST-augmented eight-class formulations. This agrees with the previous myoelectric control literature showing that compact time-domain features can be effective for movement classification when signal conditions are relatively stable and calibration is subject-specific [4]. The inclusion of cross-channel descriptors was also important for this application because neck–shoulder commands depend not only on signal amplitude but also on the distribution of activity across muscles and sides. Prior EMG work on neck muscle activity has reported symmetry-related characteristics during basic motion patterns, supporting the use of bilateral pattern and symmetry descriptors in the feature set [19].

### 4.3. Evaluation Design, Leakage Control, and Subject-Dependent Pooling

Trial-wise grouping in the pooled evaluation addressed an important limitation of overlapping-window pipelines. Neighboring windows from the same repetition can be highly correlated, so conventional window-level splitting may place very similar samples in both training and test folds and produce optimistic accuracy estimates [20]. Although pooled trial-wise grouped cross-validation remained subject-dependent, it provided a more conservative estimate than window-level splitting because all windows from the same trial were kept within the same fold, consistent with leakage-control strategies used in EMG classification studies [21]. Reporting both trial-wise grouped results and LOSO results therefore improves transparency by separating subject-dependent pooled performance from unseen-subject generalization.

### 4.4. Cross-Subject Generalization and Sources of Variability

LOSO performance was lower than within-subject and pooled subject-dependent estimates, indicating that inter-subject variability remains a major limitation to calibration-free deployment. This is consistent with the broader understanding that EMG feature distributions can shift due to factors such as differences in electrode placement and orientation and session-to-session changes. Foundational placement recommendations emphasize the importance of consistent electrode positioning and orientation to improve reliability [22]. More recent work explicitly targets electrode-shift sensitivity and proposes normalization strategies to mitigate its impact, reinforcing that robustness to placement variation is a central challenge for practical EMG interfaces [7]. In this context, LOSO results can be viewed as a baseline for “zero-calibration” use and motivate strategies that reduce calibration burden while improving transfer across users.

The reduction in LOSO performance may reflect inter-subject differences in muscle recruitment strategy, co-activation patterns, neck and shoulder movement execution, body size, skin–electrode contact, electrode placement, and IMU reference posture. The compact four-channel UT/SCM configuration was selected to support a wearable and practical sensor layout, but it may not capture all antagonist, synergistic, and accessory muscle activity involved in neck–shoulder movement. Therefore, future work should investigate whether broader neck–shoulder sEMG coverage, co-activation features, adaptive normalization, domain adaptation, or few-trial user-specific fine-tuning can improve unseen-user performance while preserving practical usability.

### 4.5. Role of IMU Fusion and Rotation Sensitivity

Combining IMU-derived kinematic cues with sEMG features is well motivated in the sensor-fusion literature because inertial signals can provide movement context when EMG patterns alone are ambiguous [1]. In this study, baseline-referenced IMU descriptors and an SCM yaw-range rotation indicator were included to represent rotation-related movement content and to support separation between shrug-only and rotation-containing commands. These features were particularly relevant for compound movements, where shoulder-elevation activity could overlap with rotation-plus-shrug commands. However, the benefit of IMU information was not uniform across all validation settings, which suggests that the contribution of inertial features depends on the command type, sensor placement, and evaluation design.

The IMU-only analysis provides an additional reference for interpreting the contribution of inertial information. Its lower performance compared with the EMG-only and EMG+IMU configurations indicates that the selected commands were not explained by inertial information alone. Instead, IMU-derived features provided complementary movement-related information alongside the sEMG activation features.

The SCM yaw-range ablation further supports this interpretation. Removing the SCM yaw-range indicator produced only small and configuration-dependent changes in accuracy, indicating that this feature provided supportive kinematic information but was not the dominant driver of classification performance. Therefore, the benefit of IMU-derived features should be interpreted in relation to the full feature set and validation design, rather than attributed to a single rotation descriptor. The tri-modal example in Figure 7 was used only as a qualitative quality-control visualization to assess cross-modal consistency after the predefined trimming and baseline-referencing steps. It was not intended to estimate physiological onset latency between muscle activation and kinematic movement, and it should not be interpreted as evidence that kinematic changes preceded EMG activation.

### 4.6. REST False Activation and Deployment Implications

For assistive control, accuracy alone is not sufficient because unintended activations during rest may affect usability and safety depending on the application. Including REST as an explicit class enabled direct estimation of REST false activation rate (FAR). In this study, the variation in REST FAR across LOSO configurations showed that the most accurate model was not necessarily the model with the lowest false activation rate. This finding supports reporting FAR, or an equivalent idle-state safety metric, alongside accuracy when selecting models for deployment-oriented assistive interfaces.

The REST results should also be interpreted cautiously because REST data were obtained from healthy participants under controlled laboratory conditions. Healthy participants can usually maintain a stable seated posture, relax the neck–shoulder muscles, and follow timing instructions consistently. In target clinical users, REST may be less well defined because of involuntary activity, increased muscle tone, spasticity, fatigue, compensatory movements, or difficulty maintaining posture. Therefore, a low REST FAR in healthy participants does not necessarily guarantee safe idle-state behavior in clinical populations. Future studies should evaluate REST FAR under more realistic resting conditions and in target users.

### 4.7. Error Modes and Confusion Patterns

The confusion matrices showed that most errors occurred between commands sharing similar activation components, particularly between shrug-only and rotation-plus-shrug classes. This pattern may be explained by the dominance of shoulder-elevation activity in the EMG envelope, while the rotational contribution may be weaker or more variable across participants. In the LOSO evaluation, laterality-dependent errors were also more evident, including left–right confusions and weaker generalization for shrug L in several held-out subjects. This suggests that inter-subject differences in muscle recruitment, movement execution, and sensor placement can affect the transferability of side-specific commands.

In the REST-augmented eight-class formulation, REST misclassification as movement contributed directly to REST FAR. Therefore, the confusion results support the need to interpret overall accuracy together with false-activation behavior, especially when considering assistive deployment. These findings also suggest that future systems may benefit from subject-specific calibration, adaptive thresholds, or post-processing strategies to reduce false activations and improve consistency across users.

### 4.8. Limitations and Future Directions

This pilot study has several limitations. First, the cohort size was small and included only young healthy male participants with a similar age range. This limits the stability of cross-subject estimates and restricts interpretation across sex, age, body size, and clinical populations. Therefore, the present findings should be interpreted as controlled laboratory feasibility rather than generalizable clinical performance. Future studies should include larger and more diverse cohorts, including female participants, older adults, and target clinical users.

Second, the selected command set consisted of relatively distinguishable movements that required clear voluntary neck–shoulder activation. This was appropriate for an initial feasibility study, but it does not establish usability in individuals with limited strength, restricted range of motion, fatigue, spasticity, or difficulty producing consistent movements. For practical assistive control, the command vocabulary may need to be adapted to each user’s preserved motor abilities. Future work should evaluate reduced-amplitude movements, patient-specific command selection, and clinical populations.

Third, the protocol used controlled seated posture, structured movement timing, and a structured recording order. Participants were instructed to begin from a neutral head/neck posture and minimize trunk motion. These controls reduced variability during data collection, but they do not represent all real-world use conditions. Torso motion, altered initial posture, unintended head tilt, fatigue, and natural postural adjustments may alter SCM, UT, IMU, and MoCap patterns and increase class confusion or false activations. Future studies should test robustness under variable posture, head tilt, trunk motion, longer sessions, and fatigue-related changes.

Fourth, the command trials were performed using fixed timing and repeated contraction/rest periods. The present study did not systematically test shorter or longer contractions, variable waiting times, or fully self-paced command initiation. Therefore, the results should be interpreted as offline window-based classification under controlled timing, not as evidence of asynchronous real-time control. Future work should evaluate self-paced commands, variable movement duration, continuous decoding, rejection of unintended movements, and recovery from false activations.

Fifth, MVC normalization was based on separate resisted MVC recordings. Although this kept normalization independent from the command trials, the resistance was not instrumented using a dynamometer. Future studies may compare resisted MVC, instrumented MVC, functional MVC, adaptive normalization, and daily recalibration strategies to determine which approach is most stable across users and sessions.

Finally, the analysis was performed offline, and the command set was not tested in a real-time closed-loop assistive device. Although the 250 ms windows, 125 ms update interval, time-domain features, simple IMU descriptors, and classical classifiers are compatible with future real-time implementation, embedded computational benchmarking and online closed-loop testing were not performed. Future work should evaluate latency, real-time stability, day-to-day repeatability, electrode reattachment, calibration burden, and online usability.

## 5. Conclusions

This pilot study evaluated a compact neck–shoulder sEMG–IMU interface for discrete assistive command decoding, with MoCap used as an external kinematic reference for movement-execution quality control. Subject-specific decoding achieved high performance for both the seven-class and REST-augmented eight-class formulations, whereas trial-wise grouped validation and LOSO testing provided more conservative estimates of generalization. The reduction in LOSO performance indicates that inter-subject variability remains a major barrier to calibration-free use. Including REST enabled safety-relevant idle-state behavior to be quantified using REST false activation rate, showing that accuracy alone is insufficient for selecting deployment-oriented models. Taken together, these findings provide a controlled offline pilot benchmark for compact neck–shoulder sEMG–IMU command decoding and support future validation in larger, more diverse, and clinically relevant populations.

## Figures and Tables

**Table 1:** Within-subject decoding performance using the best configuration for each participant in the seven-class formulation (C7) and REST-augmented eight-class formulation (C8).

Subject	C7 (7-Class)			C8 (REST-Augmented 8-Class)		
	Setup	Model	Acc (%)	Setup	Model	Acc (%)
S01	EMG+IMUfilter	LDA	96.42	EMG+IMUfilter	LDA	96.18
S02	EMG	kNN	98.77	EMG	kNN	99.29
S03	EMG+IMU	LDA	97.92	EMG+IMU	LDA	97.87
S04	EMG+IMU	LDA	97.37	EMG+IMU	LDA	97.71
S05	EMG	LDA	89.82	EMG	LDA	89.52
S06	EMG+IMU	kNN	95.81	EMG+IMU	kNN	97.52
Mean ± SD			96.02 ± 3.21			96.35 ± 3.49
Highest			98.77			99.29

Note: Best denotes the highest accuracy obtained for each participant and class setting across the evaluated modes and classifiers.

**Table 2:** Subject-dependent pooled evaluation comparing window-level stratified fivefold cross-validation and trial-wise grouped fivefold cross-validation for the seven-class formulation (C7) and REST-augmented eight-class formulation (C8).

Evaluation	Class Setting	Best Mode	Best Model	Accuracy (%)
Window-level stratified 5-fold CV (pooled windows)	C7 (7-class)	EMG	kNN	96.92 ± 0.55
Window-level stratified 5-fold CV (pooled windows)	C8 (REST-augmented 8-class)	EMG	kNN	97.08 ± 0.33
Trial-wise grouped 5-fold CV (pooled data; group = trialID)	C7 (7-class)	EMG+IMU	kNN	92.08 ± 1.05
Trial-wise grouped 5-fold CV (pooled data; group = trialID)	C8 (REST-augmented 8-class)	EMG+IMU	kNN	90.51 ± 1.93

Note: Values are reported as mean ± standard deviation across folds for the best-performing mode and classifier in each evaluation setting.

**Table 3:** Configuration-level comparison of feature modes and classifiers. Accuracy is reported as mean ± SD (%) for EMG-only, IMU-only, EMG+IMU, and EMG+IMUfilter modes using LDA, kNN, and SVM classifiers. REST FAR is reported only for the REST-augmented eight-class formulation and corresponds to the best classifier in each row.

Validation	Class Setting	Feature Mode	LDA Accuracy (%)	kNN Accuracy (%)	SVM Accuracy (%)	Best Configuration	REST FAR of Best (%)
Trial-wise grouped CV	7-class	EMG	80.79 ± 2.47	91.63 ± 2.23	86.23 ± 3.31	kNN	—
Trial-wise grouped CV		IMU-only	33.91 ± 4.80	41.90 ± 4.77	37.35 ± 4.64	kNN	—
Trial-wise grouped CV		EMG+IMU	82.00 ± 3.42	92.08 ± 1.05	88.01 ± 2.26	kNN	—
Trial-wise grouped CV		EMG+IMUfilter	78.90 ± 5.09	90.14 ± 1.92	85.83 ± 4.64	kNN	—
Trial-wise grouped CV	8-class	EMG	83.06 ± 0.87	90.32 ± 2.20	88.53 ± 2.13	kNN	0.00 ± 0.00
Trial-wise grouped CV		IMU-only	33.88 ± 3.84	44.94 ± 1.06	39.50 ± 3.82	kNN	26.04 ± 10.34
Trial-wise grouped CV		EMG+IMU	84.81 ± 1.52	90.51 ± 1.93	89.64 ± 1.93	kNN	0.00 ± 0.00
Trial-wise grouped CV		EMG+IMUfilter	83.31 ± 1.18	89.05 ± 2.17	88.05 ± 2.73	kNN	0.00 ± 0.00
LOSO	7-class	EMG	54.43 ± 11.38	50.73 ± 14.69	59.14 ± 7.53	SVM	—
LOSO		IMU-only	24.26 ± 4.71	23.43 ± 3.93	29.85 ± 7.11	SVM	—
LOSO		EMG+IMU	50.07 ± 9.86	53.18 ± 14.07	59.09 ± 5.01	SVM	—
LOSO		EMG+IMUfilter	55.99 ± 7.76	48.89 ± 14.06	60.43 ± 5.41	SVM	—
LOSO	8-class	EMG	57.25 ± 14.66	55.47 ± 15.73	60.54 ± 10.85	SVM	25.62 ± 39.62
LOSO		IMU-only	28.47 ± 3.88	28.20 ± 2.65	33.92 ± 5.16	SVM	14.17 ± 13.29
LOSO		EMG+IMU	55.49 ± 11.39	57.57 ± 15.12	62.11 ± 9.47	SVM	19.58 ± 38.08
LOSO		EMG+IMUfilter	62.06 ± 10.25	57.04 ± 15.83	63.78 ± 9.06	SVM	24.58 ± 37.03

**Table 4:** Leave-one-subject-out (LOSO) cross-subject evaluation for the seven-class formulation (C7) and REST-augmented eight-class formulation (C8).

Class Setting	Best Mode	Best Model	Accuracy (%)	REST FAR (%)
C7 (7-class)	EMG+IMUfilter	SVM	60.43 ± 5.41	-
C8 (REST-augmented 8-class)	EMG+IMUfilter	SVM	63.78 ± 9.06	24.58 ± 37.03

Note: Accuracy values are reported as mean ± standard deviation across held-out participants (*N* = 6). REST FAR is reported only for the C8 setting.

**Table 5:** Ablation analysis of the SCM yaw-range indicator. The table compares classification accuracy with and without the SCM yaw-range feature for selected trial-wise grouped cross-validation and LOSO configurations. Changes are reported in percentage points.

Validation	Class Setting	Configuration	With SCM Yaw-Range (%)	Without SCM Yaw-Range (%)	Change
Trial-wise grouped CV	C7	EMG+IMU kNN	92.08	91.70	−0.38
Trial-wise grouped CV	C8	EMG+IMU kNN	90.51	90.21	−0.30
LOSO	C8	EMG+IMU SVM	62.11	61.48	−0.63
LOSO	C8	EMG+IMUfilter SVM	63.78	63.78	0.00

## Data Availability

The data are not publicly available due to privacy and ethical restrictions related to participant recordings.

## Abbreviations

The following abbreviations are used in this manuscript:

sEMG	Surface electromyography
EMG	Electromyography
IMU	Inertial measurement unit
MoCap	Motion capture
UT	Upper trapezius
SCM	Sternocleidomastoid
MVC	Maximum voluntary contraction
%MVC	Percent maximum voluntary contraction
REST	Rest/idle class
FAR	False activation rate
LOSO	Leave-one-subject-out
CV	Cross-validation
LDA	Linear discriminant analysis
kNN	k-nearest neighbors
SVM	Support vector machine
ECOC	Error-correcting output codes
QC	Quality control
MAD	Median absolute deviation
RMS	Root mean square
MAV	Mean absolute value
WL	Waveform length
ZC	Zero crossings
SSC	Slope sign changes
NDOF	Nine degrees of freedom fusion mode
IIR	Infinite impulse response
